# The Impact of Positioning on Bottle-feeding in Preterm Infants (≤34 GA). a Comparative Study of The Semi-elevated and The Side-lying Position – a Pilot Study

**DOI:** 10.34763/devperiodmed.20192302.117124

**Published:** 2019-07-08

**Authors:** Anna Raczyńska, Ewa Gulczyńska

**Affiliations:** 1 Department of Neonatology, Polish Mother Memoriał Hospital - Research Institute, Łódź, Poland

**Keywords:** premature, orał feeding, bottle feeding, positioning, side-lying position, semi-elevated, noworodek urodzony przedwcześnie, karmienie doustne, karmienie butelką, pozycjonowanie, pozycja boczna, pozycja klasyczna

## Abstract

**Objective:**

The aim of the study was to compare the advantages of semi-elevated and side-lying positioning during bottle-feeding of preterm infants ≤34 weeks gestational age (34+0/7).

**Material and methods:**

The study included six neonates (n=6) bom ≤34 weeks gestational age who reached the age ≥32 weeks of postmenstrual age on the day when the study began and were hospitalized in the neonatology ward. Four bottle-feeding sessions were tested in each of the newborns: two in the side-lying and two in the semi-elevated position. The position for the first test was chosen randomly. For each of the positions twelve feeding sessions were examined and each preterm infant hod bottle-feeding sessions analyzed both in the semi-elevated and side-lying positions. The level of saturation and heart rate were measured as the parameters indicative of the newborn's physiological stability. The factors determining the qualitative aspect of feeding included the level of the newborn's alertness and the occurrence of choking episodes. The amount of food consumed and the duration of the feeding were also recorded.

**Results:**

The side-lying position was more effective with regard to the totol amount of sustenance consumed as compared to the semi-elevated feeding position and the study result was statistically significant (p=0.007). The difference in the number of chokes between the study groups was not statistically significant, although a trend towards a reduced number of choking episodes was observed among infants fed in the side-lying position (p=0.090). There were no significant differences in oxygen saturation, heart rate and level of activity between the study groups.

**Conclusions:**

The effects of this pilot study demonstrate the efficacy of the side-lying feeding position regarding the final amount of milk intake. The side-lying position may also reduce the number of choking episodes during the feeding. The results suggest the need to extend the study in order to confirm the potential benefits of using the side-lying position.

## Streszczenie

**Cel:**

Celem badania było porównanie korzyści związanych z zastosowaniem pozycji klasycznej i bocznej w trakcie karmienia z wykorzystaniem butelki u noworodków urodzonych przedwcześnie ≤34 tygodnia dąży (34+0/7).

**Materiał i metody:**

Do badania włączono sześcioro noworodków (n=6) urodzonych <34 tygodniu wieku ciążowego, które w dniu rozpoczęcia badania osiągnęły wiek ≥32 tygodni wieku menstrualnego, hospitalizowanych w oddziale neonatologicznym. U każdego noworodka badano cztery sesje karmienia z wykorzystaniem butelki: dwie w pozycji bocznej oraz dwie w pozycji klasycznej. Pozycja do pierwszego badania była wybierana losowo. W każdej z pozycji odbyło się dwanaście sesji karmienia włączonych do badania. Analizie poddano parametry określające stabilność noworodka: poziom saturacji oraz czynność serca. Uwzględniono też czynniki określające jakościowy aspekt karmienia, takie jak: poziom aktywności noworodka oraz występowanie epizodów krztuszenia. Analizie poddano również czynniki określające efektywność karmienia: objętość spożytego pokarmu oraz czas trwania karmienia.

**Wyniki:**

Całkowita objętość spożytego pokarmu była istotnie statystycznie większa w pozycji bocznej w porównaniu z pozycją klasyczną (p=0.007). Liczba epizodów krztuszenia nie różniła się statystycznie pomiędzy badanymi grupami, jednak u noworodków karmionych w pozycji bocznej obserwowano trend do redukcji epizodów krztuszenia (p=0.090). Nie było istotnych statystycznie różnic w poziomie saturacji, czynności serca oraz w poziomie aktywności w obu badanych grupach.

**Wnioski:**

Wyniki badania pilotażowego wskazują na większą efektywność pozycji bocznej w zakresie ilości spożytego pokarmu w trakcie karmienia. Pozycja boczna może również wpływać na zmniejszenie liczby epizodów krztuszenia. Wyniki sugerują konieczność poszerzenia badania w celu potwierdzenia potencjalnych korzyści z zastosowania pozycji bocznej.

## Introduction

Progress in perinatal care has resulted in the higher survivability of preterm infants. Nevertheless, such children need support of basic life functions, one of which is nutrition. Often they require gastric tube feeding. The transition from enteral (gastric tube) to oral feeding poses a particularly great challenge for babies who can be enrolled for oral feeding, i.e. ones with cardiovascular and respiratory stability. Therefore, it is conducted gradually, taking into account such factors as: the stability of life functions, the maturation of orofacial area reflexes, the increased activity of oral cavity muscles, suction-breathing-swallowing coordination [1, 2], and the development of autoregulatory mechanisms associated with the ability to maintain the activity level that enables feeding. The postmenstrual age of 32-34 weeks is usually when premature infants are most likely to begin oral feeding, mostly from a bottle (using maternal milk, milk from a milk bank, or modified milk) [3]. Later on, most infants born prematurely achieve a sufficient level of ability to initiate breast or mixed feeding. Indicating the proper and effective way of improving the quality and safety of oral feeding is a challenging issue.

One of the significant aspects which may contribute to improving the quality and safety of oral feeding in premature infants is the optimal feeding position, e.g. the semi-elevated (SEP) and side-lying (SLP) positions as well as their modifications.

The lack of complete oral feeding is often the reason for delayed discharge of newborn infants from hospital [4], which may increase the risk of nosocomial infection and raises the cost of hospitalization. Reducing the factors that adversely affect the introduction of full, effective and safe oral feeding is a very important element of neonatal care. Unfavourable conditions such as: saturation decrease, bradycardia or choking episodes lead to the newborns stress which may also add to the anxiety of the parents participating in the feeding process. Stress has an obvious detrimental effect on the development of the immature brain, causing neuronal atrophy and changes in the functioning of the hippocampus. Moreover, it affects the immune and neuroendocrine systems [5]. Stress negatively impacts the mother’s lactation, as it has an inhibitive effect on the secretion of hormones responsible for milk production [6]. Maternal stress may also upset the interaction with the child [7]. The safe introduction of oral feeding is an important stress-reducing factor for parents. A greater sense of safety and comfort, in turn, encourages the parents of prematurely born infants to try baby feeding on their own. A better quality of feeding can strengthen the parents’ sense of competence and reduce the stress which is related to discharge and taking up independent care for a prematurely born child [8].

So far, the research undertaken in this area has not given an unambiguous answer which position is better [9, 10, 11, 12]. This study is the first to have analyzed the occurrence of choking episodes in both the tested positions.

## Aim of the study

The aim of the study was to indicate the positioning that could improve the quantitative and qualitative aspects of bottle-feeding. The benefits of the SEP and SLP during bottle-feeding in premature newborns of ≤34 weeks gestational age (34+0/7) were analyzed.

## Material and methods

A sample of 6 preterm infants (n=6), gestational age ≤34 weeks, with the age of at least 32 (32+0/7) weeks of gestation reached by the day of initiating the study, hospitalized between June 2017 and July 2018 were recruited from the Department of Neonatology, Polish Mother Memorial Hospital Research Institute, Łódź, Poland.

The inclusion criteria were: circulatory and respiratory stability, readiness for oral feeding according to each child’s neurologopedic assessment. The parents agreed for their newborns to be bottle-fed at least twice during one day. The study included those prematurely born infants who were in the process of being transferred from tube feeding to full oral feeding and were orally fed 4-6 times within twenty-four hours.

The exclusion criteria were: disorders which could significantly affect the feeding performance, such as cleft lip and/or palate, facial paralysis, congenital defects of the facial skeleton. The presence of detected congenital malformations and low Apgar score (less than 5 points at any minute of the measurements) were among the exclusion criteria. Furthermore, newborns were excluded if they were administered analgesics, anticonvulsants and sedatives on the day of the test or up to 3 days prior to the tests, if their extubation had expired < 3 days or when bottle-feeding was not the parental preference. The participants’ characteristics are shown in Table I.

**Table I:** The participants' characteristics. Tabela I. Charakterystyka uczestników badania.

Analyzed feature *Analizowana cecha*	Baby 1 *Dziecko 1*	Baby 2 *Dziecko 2*	Baby 3 *Dziecko 3*	Baby 4 *Dziecko 4*	Baby 5 *Dziecko 5*	Baby 6 *Dziecko 6*
**Birth body weight** ***Urodzeniowa masa ciała***	**1750 g**	**2100g**	**1700 g**	**1500 g**	**1600 g**	**1980 g**
**Gestational age at birth** ***Wiek ciążowy***	**30**	**34**	**32+4**	**29**	**30+2**	**33+5**
**Gestational age at beginning of the study** ***Wiek ciążowy w momencie rozpoczęcia badania***	**33**	**36**	**33**	**36**	**33**	**35**
**Apgar score** ***Punktacja Apgar***	**6/6/6/6**	**9/9/9/9**	**9/9**	**6/6/7/7**	**7/7/6/8**	**7/7/7**
**Gender** ***Płeć***	**M** **S**	**F** **C**	**M** **S**	**M** **S**	**F** **C**	**M** **S**
**Neurological background** ***Podłoże neurologiczne***	**IVH II**	**None** *Brak*	**None** *Brak*	**IVH I**	**IVH I**	**None** *Brak*
**Respiratory support during the study** ***Wsparcie oddechowe w trakcie badania***	**None** **Brak**	**None** *Brak*	**None** *Brak*	**None** *Brak*	**None** *Brak*	**None** *Brak*

**Abbreviations**: *Definicja użytych skrótów*: IVH – intraventricular haemorrhage; M– male, F– female IVH – krwawienie około-dokomorowe; S– syn, C– córka

**Fig. 1:**
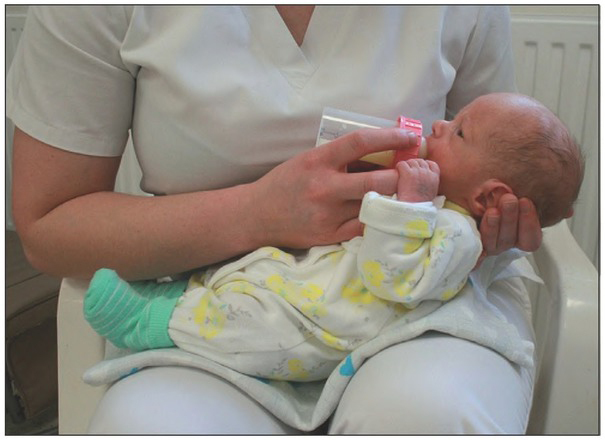
Infant fed in the semi-elevated position. Rye. 1. Dziecko karmione w pozycji klasycznej.

**Fig. 2:**
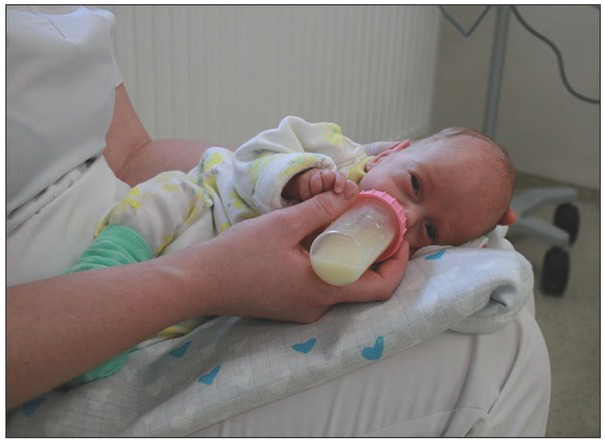
Infant fed in the side-lying position. Ryc. 2. Dziecko karmione w pozycji bocznej.

The research was designed as a crossover, alternate study. It was a randomized trial. Each infant was bottle-fed four times: twice in the SEP and twice in the SLP. In each of the study positions 12 feeding sessions were analyzed. The photographs of both positions are shown in Figure 1 and Figure 2. The position for the first trial was randomly assigned (random table), then positioning changed after each feeding session. In one day, only two consecutive feeding sessions were recorded in order to minimize fatigability as a disrupting factor. The study included the analysis of oxygen saturation (SpO_2_) level, heart rate (HR, pulse oximeter data) and level of activity 2 minutes before the feeding session as well as in the 3rd minute, the 10th minute and at the end of the feeding session. Furthermore, the volume of food eaten in the 10th minute of feeding and at the end of the feeding session was analyzed.

The level of activity was assessed according to the 6-point Neonatal Behavioural Assessment Scale (Brazelton scale) [13], where individual points mean: 1 – quiet sleep, 2 – active sleep, 3 – drowsy, 4 – quiet alert, 5 – active alert, 6 – crying.

During each feeding session, the same type of teat and bottle were used. During the study feeding, normal feeding session procedures were applied: avoidance of excessive stimuli by ensuring a calm environment with a limited level of light and noise [14], pauses in the feeding after a series of suckling (interval feeding), manual support within the external part of the mouth (chick and chin) to improve lip tightness and/or mandibular function. Stimulation of the suction reflex was also allowed.

The maximum feeding session time amounted to 40 minutes from the moment of taking the newborn from bed for feeding, including interruptions, such as pauses for burping or for equalizing the breath. The duration of each feeding in the study was calculated from the moment of teat insertion in the newborns mouth for the first time. Pauses in the feeding were excluded, i.e. the periods of feeding that were indicated referred to the time when the teat was actually in the child’s mouth.

Oxygen supplementation was possible during the study if ordered by the doctor. Each feeding session was recorded with a video camera.

The study was approved by the Bioethical Commission of the Polish Mother’s Memorial Hospital Research Institute, Łódź, Poland; opinion no. 95/2016.

## Results

### Statistical analysis

The factors that were investigated described the measures of location as mean values, along with measures of dispersion as standard deviation, a 95% confidence interval, and minimum-to-maximum values.

A multifactor analysis of variance was performed for statistical analyses of oxygen saturation, heart rate and percent intake, incorporating both repeated measurements for the two separate feeding positions and differences between the two investigated positions. An ordinal regression was carried out for the analysis of the infants’ activity level, as above, considering both repeated measurements and the differences between the two feeding positions. Poisson regression considering the discrepancies between the groups was fitted for the analysis of the number of chokes. The value of p<0.05 was considered statistically significant. All the statistical computations were carried out by means of Stata/Special Edition, release 14.2 (StataCorp LP, College Station, Texas, USA).

### Oxygen saturation

**Fig. 3:**
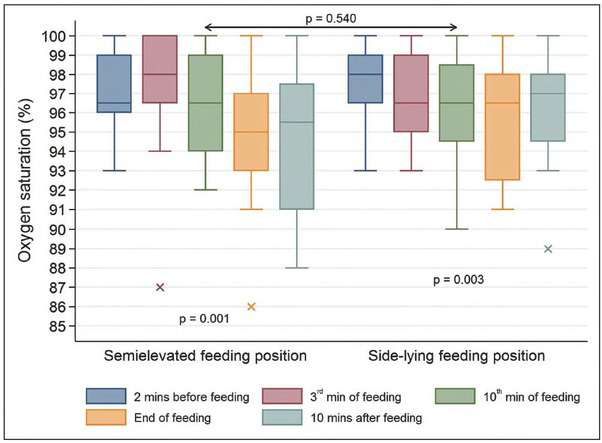
Oxygen saturation (%) in the study infants. Rye. 3. Poziom saturacji (%) u badanych dzieci.

Oxygen saturation fluctuations were statistically significant in relation to the initial values both in the infants fed in the SEP (p=0.001) and those fed in the SLP (p=0.003). There was no significant difference between the two study groups (p=0.540). Considering the SEP, the highest mean value was observed in the third minute of the feeding (97.17% ± SD = 3.69%), while the lowest value was recorded 10 minutes after the feeding (94.25% ± SD = 4.24%). When analyzing the SLP feeding, the highest mean value was noted two minutes before the feeding (97.50% ± SD = 2.02%), while the lowest one was recorded at the end of the feeding (65.67% ± SD = 2.99%). Graphic distributions of this variable are shown in Figure 3.

### Heart rate

**Fig. 4:**
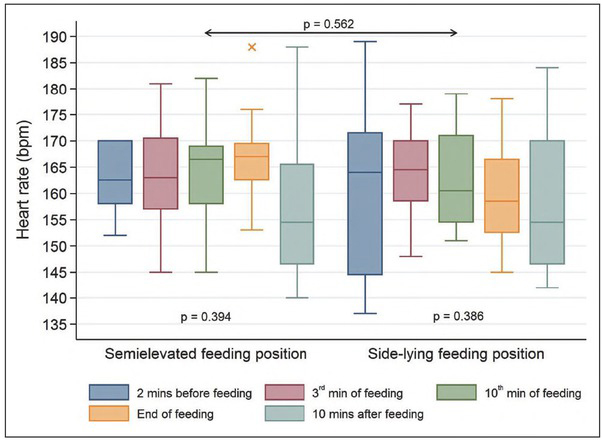
Heart rate (bpm) in the study infants. Ryc. 4. Czynność serca (uderzenia na minutę) u badanych dzieci.

The changes in heart rate were not statistically significant in the infants fed in the SEP (p=0.394) and those fed in the SLP (p=0.386). It is noteworthy that in the SEP feeding, the largest mean value was observed at the end (166.83/bpm ± SD = 9.53/bpm) and the lowest one was recorded 10 minutes after completion (156.75/bpm ± SD = 14.86/bpm). For the SLP, the highest mean value was observed in the third minute of the feeding (164.08/bpm ± SD = 8.90/bpm), while the lowest one was detected ten minutes afterwards (158.25/bpm ± SD = 15.23/bpm). There was no significant difference between the two study groups (p=0.562). The graphic distribution of this variable is shown in Figure 4.

### Level of activity

**Fig. 5:**
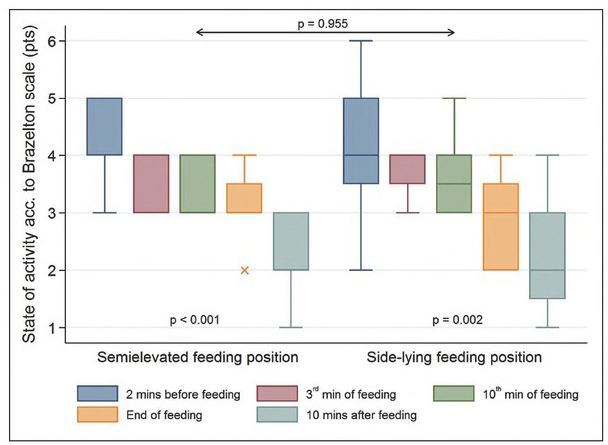
The studied infants' level of activity according to the Brazelton scale (pts). Ryc. 5. Stan aktywności badanych dzieci wg skali Brazeltona (pkt).

The study infants’ level of activity (according to the Brazelton scale) was decreasing constantly. The result was statistically significant during the observation, both in the study participants fed in the SEP (p<0.00l) and those fed in the SLP (p=0.002). There was no statistically significant difference between the two study groups (p=0.955). For the SEP, the highest mean value was noted two minutes before the feeding (4.50 ± SD = 0.67), while the lowest one was observed 10 minutes afterwards (2.25 ± SD = 0.75). Considering the SLP, the highest mean value was also recorded two minutes before the feeding (4.17 ± SD = 1.11), while the lowest level was observed ten minutes after it finished (2.33 ± SD = 1.07). The graphic distribution of this variable is shown in Figure 5.

### Amount of food intake

**Fig. 6:**
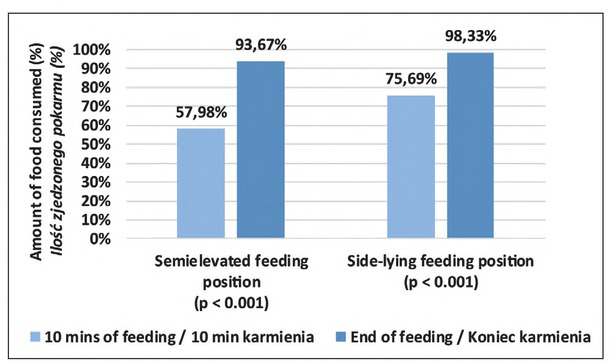
Amount of food consumed, computed as the amount observed *versus* the amount expected (%). Ryc. 6. Ilość spożytego pokarmu obliczona jako stosunek ilości obserwowanej do oczekiwanej (%).

On average the study infants fed in the SEP consumed 57.98% of the sustenance in the tenth minute and 93.67% at the end of the feeding (p<0.001), as recommended by neonatologists. The study infants fed in the SLP consumed an average of 75.69% of the formula in the tenth minute and 98.33% at the end of the feeding (p<0.001). The SLP was more effective with regard to the total amount of sustenance the infants had consumed when compared with the SEP (p=0.007) and the difference was statistically significant. The graphic distribution of this trait is shown in Figure 6.

### Other factors

There was no statistically significant difference between the study groups in feeding duration (p=0.198). All the infants (n=6) completed their feeding within 40 minutes of the feeding session.

Regarding the number of choking episodes, the result of studying the two groups was not statistically significant, either (p=0.090), although the infants fed in the SEP choked apparently more frequently than the infants fed in the SLP. The weighted means for SEP (M=0.75) and for SLP (M=0.33) were calculated on the basis of the number of choking episodes that occurred in a given feeding position. At the 12th feeding session, the choking episode occurred 9 times in the SEP, and 5 times in the SLP. Descriptive statistics of this variable are shown in Table II.

**Table II:** Descriptive statistics of chocking episodes in the studied infants. Tabela II. Statystyka opisowa dla epizodów krztuszenia u badanych dzieci.

Analyzed factor *Analizowana cecha*	Feeding position *Pozycja karmienia*	Statistical parameters *Parametry statystyczne*				Level of statistical significance *[p-value]* *Poziom istotności statystycznej*
		M*	*SD’*	95% Cl**	*Min. – max*.	
**Choking (number of events)** **Krztuszenie (*liczba* zdarzeń)**	**Semi-elevated** ***Klasyczna***	**0.75**	**0.62**	**0.36-1.14**	**0-2**	**= 0.090**
	**Side-lying** ***Boczna***	**0.33**	**0.49**	**0.02-0.65**	**0-1**	

*M – mean (weighted mean); †SD – standard deviation; **95% Cl – confidence interval *M – średnia (średnia ważona); ’SD – odchylenie standardowe; **95% Cl – przedział ufności

## Discussion

Identification of a more efficient feeding position which leads to increased food intake would make it possible to reduce the need for gastric tube feeding and, consequently, the risk of its adverse effects (e.g. desaturations, irritation of the mucosa, esophageal perforation and other iatrogenic traumas). It is common for prematurely born babies not to eat a full portion. In the majority of such cases, gastric tube feeding is required or considered in neonatological practice, so that the full portion is provided and the neonates properly gain weight.

Increased milk intake was observed in the SLP in comparison to the SEP within a similar period of time, so the SLP appears more beneficial in terms of the efficiency of feeding. Dawson *et al*. [9] compared bottle-feeding in cradle hold and side-lying positions in infants (n=25) with a slightly greater maturity (34 postmenstrual age). Dawson *et al*. also noticed this relationship: the infants tended to consume a smaller portion of milk in cradle hold compared with the side-lying position, the mean (SD) was 82% (25) versus 87% (20), respectively (P=0.08, 95% CI -0.64, 10.00). The amount of food eaten by newborns in the present study fed in SLP both at the 10th minute of feeding and at the moment of termination of feeding was higher, which indicates higher efficiency of SLP.

Since the readiness for oral feeding and the efficiency of feeding is also related to the level of activity [15], newborns participating in the study were evaluated according to the Brazelton scale (NBAS) [13]. Between the two study groups there was no statistically significant difference (p=0.955). The Brazelton scale, however, has limitations when applied in premature newborns, therefore, the present study used the scale as an auxiliary tool.

The ability to self-feed is essential for planning the baby’s discharge from hospital. From the authors’ observations and parents’ comments it can be concluded that the fear of choking due to incorrect feeding is the main cause of feeding-related anxiety. The present study is the first to have collected data on the number of episodes of choking while feeding. Choking is relatively common when newborns are fed and choking episodes are extremely traumatic both for parents and their newborns. Choke occurrence may be related to various factors. The most common causes include suction-swallowing-breathing disorders and problems with oral transfer due to improper lip tightness during suction. The proper selection of a bottle teat, sized and shaped to the size of the child’s mouth, is also important. If the milk flow in the teat is too fast, it also increases the risk of aspiration due to choking episodes [16], because preterm infants have limited ability to self-regulate milk flow [17]. For this reason, a teat with the smallest possible flow should be used to increase the safety of feeding. In the present study, only one type of 50ml bottle and teat appropriate for infants born prematurely was used for feeding in order to maintain the same conditions of milk outflow and its slowness in each feeding.

Although choking episodes are not a real threat to the health of a prematurely born infant, they may cause desaturation, apnea or bradycardia. These are also undesirable feeding-related events, which reduce the involvement of prematurely born infants in the feeding process, thereby prolonging the time before satisfactory oral feeding is achieved [18]. Moreover, choking episodes may increase the risk of aspiration pneumonia. That is why it is so important to indicate the position for oral feeding, which will result in less frequent episodes of choking.

It seems that the SLP promotes a greater relaxation of the newborn’s muscles because of the larger support plane and less compression on the peritoneal cavity (indirectly affecting the diaphragm and the pleural cavity). Therefore, it can improve the effectiveness of respiratory muscles and structures involved in feeding by reducing the impact of other structures interacting indirectly.

The less frequent occurrence of choking episodes renders the SLP safer. This may also be related to the horizontal arrangement of the bottle (SLP) in which the outflow of milk may be slower than in the vertical position (SEP). In the vertical positioning of the bottle, the force of gravity promotes a faster outflow of milk. Because one type of bottle and nipple were used in the study, the slower flow of milk in the SLP could be considered an additional mechanism to slow the outflow of milk dependent on gravity. The proper selection of the feeding position will help to minimize direct negative factors in oral feeding performance. The best positioning can also provide potential benefits in infancy and early childhood, as it helps to reduce distant consequences, i.e. neonatal onset feeding disorders. The studies available indicate that prematurely born babies are particularly predisposed to early childhood feeding disorders [19, 20, 21].

It is noteworthy that in all the previous studies on a similar subject the study newborns were bottle-fed in the SLP in its different variants [9, 10, 11, 12]. Therefore, in the cases described there, although the newborns lay on the side, there were differences in their lateral positions. Such differences may be of some significance if a potential influence of indirect factors on lateral position feeding is taken into account. The largest difference in lateral positioning was found in the study by Park et al. [12], where the lateral position was defined as the semielevated side-lying. In such a feeding strategy, the lateral position seems to be less conducive to muscle relaxation, because the shoulder girdle is raised and the newborns upper body does not have a large supporting plane as was the case in the lateral position used in the present study.

The mothers of newborns who were involved in this study were encouraged to breastfeed and informed about the pro-health effects of breastfeeding for mother and child [22]. In neonatal practice, bottle-feeding is used as a temporary solution [3] until full breastfeeding is achieved, or if breastfeeding is impossible for any reason.

## Limitations

Above all, the number of participants is the most important limitation of this study. Although the number of the feeding sessions that were tested made it possible to perform statistical analysis, the results obtained require confirmation, as does the hypothesis that the SLP may reduce the number of choking episodes. In the above study, it was only possible to show that the episodes of choking were less frequent in the SLP (trend). Therefore, further research including a larger study group is necessary.

## Conclusions

The best feeding position for premature babies is yet to be determined. However, the SLP has been proven to be safe when introducing oral feeding in premature babies. The amount of food consumed by newborns during and at the end of feeding was greater, and the difference was statistically significant. This initially indicates its higher effectiveness. Also, a smaller number of choking episodes may indicate greater safety of the lateral position when feeding premature babies. Since the study was conducted on a small number of participants, it has its limitations. Future trials on a larger sample will make it possible to determine the benefits of the SLP and its advantages in a more precise way.
